# A Manual of Dental Mechanics

**Published:** 1874-11

**Authors:** 


A MANUAL OF DENTAL MECHANICS. By Oakley Cales.

*Licentiate of Dental Surgery of the Royal College of Surgeons, Dental Surgeon to the Hospital of Diseases for the Throat.* With one hundred and fifty illustrations.

This is a hand-book for the student, containing matter of a purely practical nature, so arranged as to carry the student along in the course by easy consecutive steps.

It takes up and presents in a familiar manner all the processes and modes in the construction of artificial dentures in the various styles now employed.

The various modes of inserting pivot teeth are very clearly described, so that any one possessing any familiarity with the subject could readily follow out the proposed method.

Section twelve treats of deformities of the mouth, especially cleft palates and the method of remedying such defects, at least so far as it can be done with mechanical appliances.

There is also an appendix, containing valuable information in the way of practical suggestions, formulas, etc. The work we regard as a very valuable one to the student, and indeed for the practitioner. It should be in every dentist's library. It is reprinted in this country by Lindsay & Blakiston, and may be obtained of any bookseller who keeps medical work, price 2.50

